# Effects of focal metallic implants on opposing cartilage – an in-vitro study with an abrasion test machine

**DOI:** 10.1186/s12891-020-03292-4

**Published:** 2020-04-21

**Authors:** Theresa Diermeier, Arne Venjakob, Kevin Byrne, Rainer Burgkart, Peter Foehr, Stefan Milz, Andreas B. Imhoff, Stephan Vogt

**Affiliations:** 1 Department of Orthopaedic Sports Medicine, Klinikum rechts der Isar, Technische Universität München, Ismaninger Str. 22, 81675 Munich, Germany; 2 Department of Rheumatology and Arthroscopy, Marienkrankenhaus Düsseldorf-Kaiserswerth, Düsseldorf, Germany; 3 Department of Orthopaedics, University of Pittsburgh, Pittsburgh, PA USA; 4 Department for Orthopedics and Orthopedic Sports Medicine, Klinikum rechts der Isar, Technische Universität München, Munich, Germany; 5 Department of Anatomy Munich, Ludwig-Maximilians University Munich, Munich, Germany; 6 Department of Sports Orthopedics, Hessing Klinik, Augsburg, Germany

**Keywords:** Cartilage, Knee, Focal metallic implant, Hemicap

## Abstract

**Background:**

For focal cartilage defects, biological repair might be ineffective in patients over 45 years. A focal metallic implant (FMI) (Hemi-CAP Arthrosurface Inc., Franklin, MA, USA) was designed to reduce symptoms. The aim of this study was to evaluate the effects of a FMI on the opposing tibial cartilage in a biomechanical set-up. It is hypothesized that a FMI would not damage the opposing cartilage under physiological loading conditions.

**Methods:**

An abrasion machine was used to test the effects of cyclic loading on osteochondral plugs. The machine applied a compressive load of 33 N and sheared the samples 10 mm in the anteroposterior direction by 1 Hz. Tibial osteochondral plugs from porcine knees were placed in opposition to a FMI and cycled for 1 or 6 h. After testing each plug was fixed, stained and evaluated for cartilage damage.

**Results:**

After 1 h of loading (*n* = 6), none of the osteochondral plugs showed histologic signs of degradation. After 6 h of loading (n = 6) three samples had histologic signs of injury in the tangential zone (grade 1) and one had signs of injury in the transitional and deep zones (grade 2). Exploration for 6 h resulted in significant more cartilage damage compared to the shorter exploration time (*p = 0.06).* However, no significant difference between saline and hyaluronic acid was evident (*p = 0.55*).

**Conclusion:**

Under physiologic loading conditions, contact with a FMI leads to cartilage damage in the opposing articular cartilage in six hours. In clinical practice, a thorough analysis of pre-existing defects on the opposing cartilage is recommended when FMI is considered.

## Background

Chondral pathology is commonly encountered in the knee, with 61–66% of knee arthroscopy procedures reporting chondral or osteochondral lesions [11, 23, 41]. In patients over 40 years, the medial compartment is most commonly affected [3, 11]. Surgical treatment options vary considerably based on the size and locality of the lesion. Smaller lesions, generally under 2 cm in diameter [30], may be treated by debridement, microfracture [38, 39] or, if indicated, osteochondral autograft transfer [21, 27]. While good patient reported outcomes (PRO) have been demonstrated in younger patients, the aforementioned biologic techniques were found to result in reduced PRO scores in those over 45 years old [21, 26]. However, untreated cartilage defects are at risk to progress to osteoarthritis (OA) [8, 19], and severe symptomatic OA may necessitate partial or total knee arthroplasty (TKA). TKA is generally not recommended in younger patients, as there are higher rates of aseptic mechanical failure, periprosthetic joint infection, and reduced PRO scores after TKA in patients under 60 years old [18, 22, 32].

Therefore, therapeutic options in middle-aged patients with focal cartilage defects remain limited. Biologic treatment options directly addressing focal cartilage defects do not provide the same benefit seen in younger patients, while the more aggressive TKA is generally reserved for severe symptomatic osteoarthritis in older patients. A focal metallic implant (FMI) (Hemi-CAP Arthrosurface Inc., Franklin, MA, USA) was developed as a midway point specifically for such patients to treat symptomatic focal cartilage defects and prevent progression of OA. Previous studies have focused on the optimal position of the FMI in regard to joint pressure and implant behavior in animal models. While these studies demonstrated the congruence of the implant with surrounding cartilage to be significant in preserving physiologic joint pressure [4–7], other studies focused on the outcome and implant behavior of the different implant designs in various animal models [13–15, 24]. A protruded implant may be a clear etiology of damage to opposing cartilage, however some animal studies also reported defects in the setting of a *flat implant* [4, 5, 24].

The purpose of the present study was to evaluate the mechanical effects of a FMI on opposing cartilage. This tribological study was designed to simulate repetitive articulation under perfect implant conditions. It was hypothesized that, under simulated physiologic joint conditions, the opposing cartilage would not be damaged by an FMI. In recent literature hyaluronic acid was described as a boundary lubricant, which reduces the friction coefficient and therefore a beneficial effect of hyaluronic acid in present set-up was expected.

## Methods

### Specimen and study design

Fresh frozen porcine knees from a local butcher (*n* = 21, age between 6 and 7 month, body weight 80.5 ± 8.5 kg) were used for this study. After slaughtering, knees were removed from the animals and adhering soft tissue was carefully removed. Knees were stored at − 20 °C and thawed overnight at room temperature before testing. After thawing, osteochondral plugs with a diameter of 10 mm and height of 12 mm were harvested (OATS® system, Arthrex, Florida, USA) from the center of the medial tibial plate. Each of the plugs was then fixed into the abrasion machine.

In a previous study utilizing the same preparation detailed above, osteochondral plugs of the same dimensions were also harvested from the femoral condyle (OATS® system, Arthrex, Florida, USA) [40]. Afterwards the tibial osteochondral plugs were cycled against the osteochondral plugs from the opposing femoral condyle in a saline medium for 24, 48, and 72 h as a proof-of-concept (*n* = 9). Testing native femoral osteochondral plugs against native tibial osteochondral plugs does not result in macroscopic or microscopic deformation or damage to any cartilage zones even after 72 h of continuous cycling.

In the present study, the tibial osteochondral plugs were tested against a FMI (Hemi-CAP Arthrosurface Inc., Franklin, MA, USA) in an abrasion machine. The surface of the FMI contains of a chrome cobalt alloy, which is fixed to a titanium alloy screw. The plugs were randomized to a testing duration (1 or 6 h) and randomized to either a saline (NaCl 0,9%, RT) or hyaluronic acid (Viscoseal Syringe, TRB Chemedica AG, Haar/Germany) medium, to avoid effect of the medium (Table 1).

**Table 1:** Friction partners, exploration time and test mediums

Friction partners	Exploration Time (h)	Sample size	Test medium hyaluronic acid/saline
Tibial Cartilage on FMI	1	6	3/3
Tibial Cartilage on FMI	6	6	3/3

*FMI*, focal metallic implant

### Liner friction testing system

**Fig. 1:**
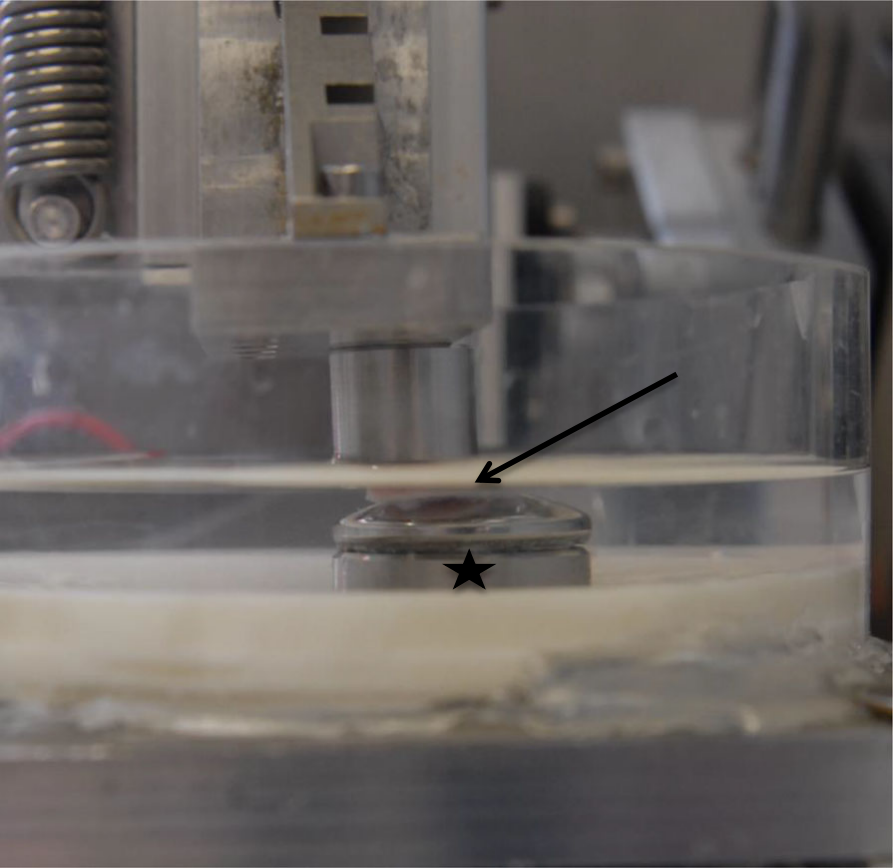
Experimental set-up with the FMI (asterix) at the bottom and the osteochondral plug (black arrow) on top

As previously described all tests were performed in a specially designed abrasion test machine (Fig. 1) [40]. In summary the system consisted of a drive mechanism connected to a mobile platform and a fixed base. The rotatory motion from an electric motor was translated into a linear agitation by use of a tappet. The distance of the linear motion was adjustable from 2 mm up to 20 mm (stroke = 40 mm), while cycle frequency was adjustable between 0.5 and 2.25 Hz. For application of an axial load to the specimens a second axis was attached perpendicular to linear axis. A force sensor (maximum load of 200 N, Type 8431–5200, Burster Gernsbach/Germany), an adjustable screw, and a spring, connected in series, were used to apply a constant axial load. Specimens could be fixed to the fixed base plates using a screw coupling. A transparent plastic cylinder was fixed to the base plate to allow the samples to be submerged in a liquid medium. Cycle frequency, number of cycles (ZX122, Motrona, Rielasingen/Germany) and applied force (tare function, actual and absolute maximal force) could all be adjusted via a control unit. Stainless steel, aluminum and polymethylmethacrylate (PMMA) were used as the only materials in contact with the specimen.

For the present study the following parameters were set: Axial load was 33 N, amplitude of linear motion was 10 mm, and cycle frequency was 1 Hz. The cycle frequency was adapted to normal walking. The load of 33 N was calculated in regard to the pig’s weight (average 80.5 kg), the articular surface of a porcine knee, and the diameter of the osteochondral plug (10 mm). The stress rate was calculated with 0.42 MPa.

### Macroscopic examination

For macroscopic evaluation all osteochondral plugs were stained with Indian ink according to the protocol set forth by Meachim [31]. While an objective macroscopic classification system was not used, the findings are discussed subjectively below. Histological scoring was found to be more appropriate for classifying the defects in detail.

### Histological examination

Fixation began by dehydrating the osteochondral plugs in increasing concentrations of ethanol and then clearing in Xylene. After an additional washing step in 100% methanol, specimens were embedded in methyl methacrylate (MMA) [33]. After complete polymerization of the MMA, the blocks were cut in the middle with an annular diamond coated saw (Leica saw microtome SP1600, Leica, Nussloch, Germany). Form the central part of each plug 150 μm thick sections were cut perpendicular to the cartilage surface, starting from the center of the osteochondral plug. Afterwards sections were glued onto plastic slides and polished to a thickness of 80 μm. The sections were then stained with Giemsa-Eosin [20]. A histological scoring system was used to assess the depth of potential defects. A defect severity score was assigned to each specimen depending on which cartilage zones were affected. For better discrimination of cartilage zones, bright field microscopy with polarized light was used. The investigator was blinded to the testing protocol of individual specimens and scored representative sections independently.

### Histologic classification

**Fig. 2:**
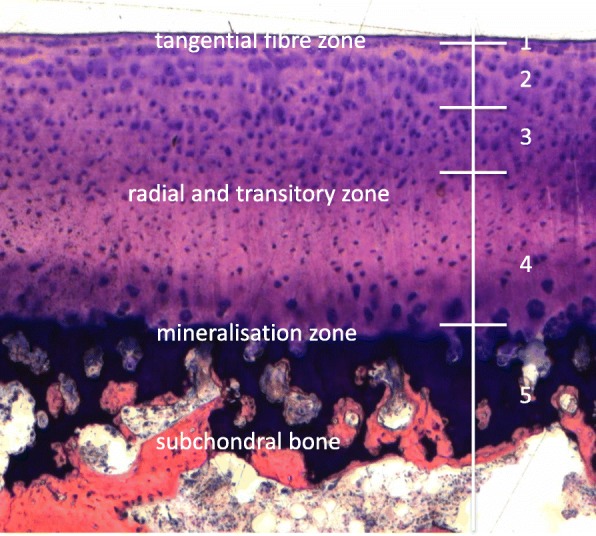
Scoring system for damage classification

The worst damage was classified according to the affected zones as follows (Fig. 2) [40]:

Score of 0 = no deformation / damage detectable; 1 = damage of only the tangential (i.e. superficial) zone of cartilage; 2 = damage of the tangential zone and up to 25% of the transitional and radial (i.e. deep) zone of cartilage; 3 = damage of the tangential zone and up to 50% of the transitional and radial (i.e. deep) zone of cartilage; 4 = damage of the tangential zone and up to 100% of the transitional and radial (i.e. deep) zone of cartilage; 5 = damage of all non-mineralized layers plus the underlying mineralized cartilage layer.

### Statistical analysis

Statistical analysis was performed by use of SPSS software (SPSS, Chicago, IL). For all statistical tests, *p* values less than 0.05 were considered as significant. For comparison between different exploration times and mediums the Fisher’s exact test was utilized.

## Results

### Macroscopic results

**Fig. 3:**
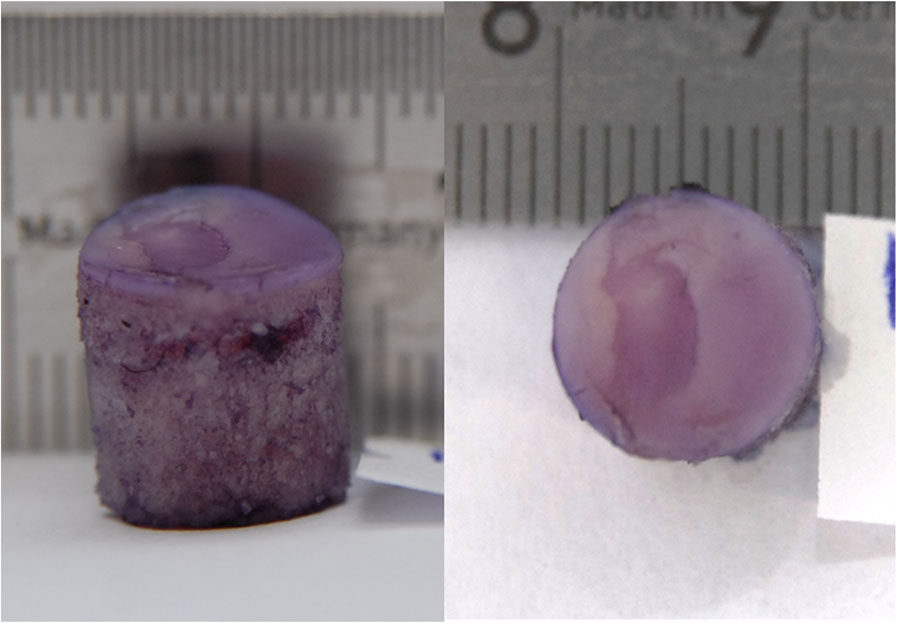
Macroscopic picture of a specimen stained with ink according to Meachim [31] after tested for 6 h in hyaluronic acid. Left picture 0° perspective; right picture 90° perspective

After staining with Indian ink, each osteochondral plug was macroscopically evaluated. After abrasion of FMI on tibial osteochondral plugs in either saline or hyaluronic acid, no macroscopic damage was noted after 1 h. However, after 6 h, signs of cartilage damage were present in one of three specimens tested in saline, and in two of three specimens tested in hyaluronic acid (Fig. 3).

### Histologic results

**Fig. 4:**
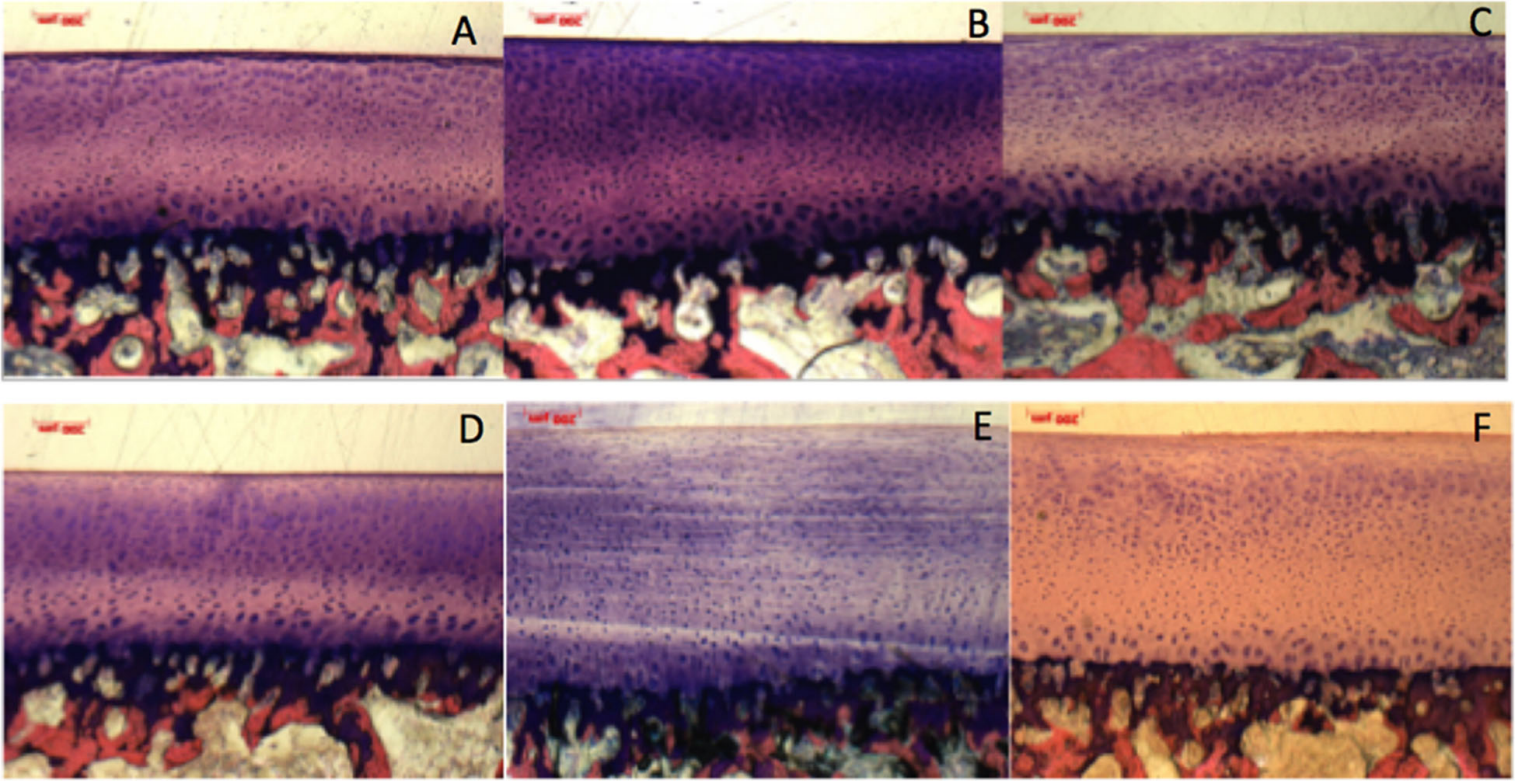
Histologic pictures of specimens tested for 1 h in saline (**a**-**c**) and hyaluronic acid (Viscoseal Syringe, TRB Chemedica AG, Haar/Germany (**d**-**e**) without any cartilage defect. (Giemsa 5x)

After abrasion of FMI on tibial osteochondral plugs in either saline or hyaluronic acid for one hour, no histologic damage was noted in any cartilage zones (Fig. 4).

**Fig. 5:**
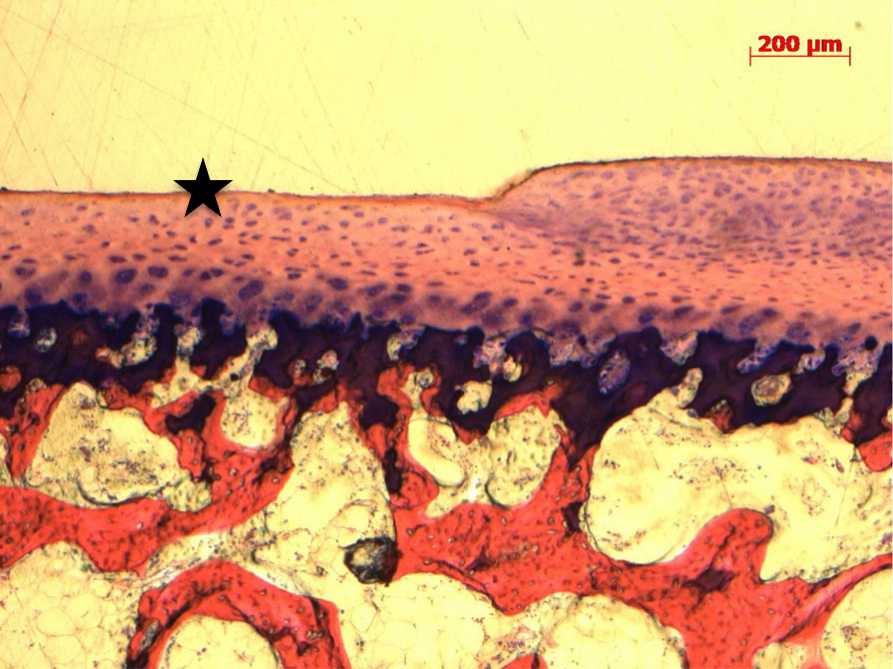
Histologic picture of a specimen tested for 6 h in hyaluronic acid (Viscoseal Syringe, TRB Chemedica AG, Haar/Germany with a defect of the upper 25% of the transitional and radial zone (grade 2) (asterix). (Giemsa 5x)

After 6 h 50% (3/6) of the specimens had a damage of the tangential zone (grade 1 – one saline, two hyaluronic acid) and 16.7% (1/6) of the plugs displayed damage to the upper 25% of the radial zone (grade 2 – hyaluronic acid) (Fig. 5.)

Overall, no statistical significant difference between saline and hyaluronic acid was evaluated (*p* = 0.55) for both exploration times. However, exploration for 6 h resulted in significant worse cartilage damage compared to 1 h (*p* = 0.06).

## Discussion

The most important finding of the present study is that cyclic loading under simulated physiologic joint pressure with optimal FMI positioning resulted in superficial defects to opposing cartilage in as little as 6 h. Furthermore, hyaluronic acid had no benefit to the opposing cartilage in this biomechanical set-up.

Recent literature has highlighted the importance of implant position in regards to surrounding cartilage. Becher et al. [4–7] analyzed various implant positions and demonstrated that 1 mm of implant protrusion resulted in an increase of peak contact pressure of up to 217% compared to native joint conditions [5]. In contrast, congruence of the implant with surrounding cartilage lead to no significant differences in static and dynamic testing conditions when compared to native cartilage [4]. Kirker-Head et al. [24] described a correlation between damage to opposing cartilage and a protruded implant position in an animal model. An increase in contact pressure and the effect of an elevated edge on an implant are therefore thought to be responsible for defects on opposing cartilage. In the present protocol, optimal implant position was simulated by applying physiological joint [17] pressure (0.42 MPa) on a FMI and the opposing tibial cartilage, eliminating concern of congruity with surrounding cartilage. Although bovine cartilage in comparison to elderly human cartilage has differences in biomechanical properties (less Young’s moduli and greater hydraulic permeability) it demonstrates similar responses to injurious loading [17]. Cartilage in porcine knees has been shown to demonstrate elastic deformation without failure for axial load up to 5 MPa [37]. For comparison, the equilibrium contact modulus in bovine knees is 0.62 ± 0.1 MPa [34]. Consequently, the applied axial stress in the present study may be considered to be well within the range of physiological values for porcine cartilage.

The function of the abrasion test machine was evaluated and verified in pre-tests. Cycling of a native tibial on a femoral osteochondral plug in a saline medium did not result in macroscopic or histologic damage even after 72 h of continuous cycling. In the present study, a saline medium was used for half of the samples. The other half were tested in hyaluronic acid, which was hypothesized to be more similar to synovial fluid and reduce friction between two articulating surfaces by working as a boundary molecule [17]. Nevertheless, cartilage damage was seen after just 6 h of cycling in both test mediums. The friction coefficient for bovine cartilage-to-cartilage (0.024 ± 0.004 [34]) is similar to that of chrome-cobalt alloys to cartilage (0,021 to 0,023 [35]), yet superficial cartilage damage was still observed. Nevertheless, further studies are needed to evaluate the effect of hyaluronic acid and the progression of the superficial defects in longer cycling times.

Although the defects in this study were created in vitro, it might be reasonable to hypothesize that such defects could also develop in vivo. The progression of untreated cartilage defects over time has been shown by several clinical and radiological studies [8, 16, 36]. Therefore, special attention should be given to the health of the tibial cartilage if a FMI is indicated. Furthermore, cartilage lesions are also often accompanied by meniscal tears [10, 23]. Various meniscal tear types, as well as partial meniscectomies, are associated with changes in peak contact pressure and contact area between articulating cartilage [1, 2, 25]. Becher et al. [6] evaluated the effect of various FMI positions on contact pressure in the setting of a complete radial tear of the medial meniscus and found significant increases of peak contact pressure even with flush implant positioning. It could therefore be hypothesized that in the setting of both pre-existing tibial cartilage defects and a torn or resected meniscus an FMI might be contraindicated. A thorough analysis of concomitant injuries is recommended when an FMI is indicated.

Several studies have investigated bony integration, effects on opposing cartilage, and defect progression of FMIs in various animal models and found heterogeneous results [9, 12–14, 24, 28, 29]. Some studies showed good bony integration of implants while other studies reported histological damages to the opposing cartilage [12, 15]. Due to the variety in animal models and used devices, the reasons for the cartilage defects remain unclear. While some authors describe a relationship between implant position and cartilage defects, other studies found cartilage defects in the setting of a flat implant position. More recently clinical results have become available, which show significantly improved patient reported outcome scores after a mean follow-up of 5.3 years [7]. Radiographs at final follow-up did show slight progression of osteoarthritic changes, but re-arthroscopy to evaluate joint surfaces was not performed. The findings of this present study, as well as existing animal studies, suggest that damage to opposing cartilage could become more obvious in longer-term clinical studies.

The present study has several limitations. First of all the data was obtained in vitro, which eliminates the complex interplay of intra-articular forces and healing factors present in living tissue. Additionally some animal studies reported an overgrowth of the superficial cartilage zone over the border of the FMI, which might have some protective effect to the opposite cartilage [24]. The abrasion test machine could also only simulate unidirectional sliding without any rotatory component, and did so in a test medium that only approximated synovial fluid. Therefore intact knee joint kinematics were only approximately simulated. Furthermore, porcine knees were used, which differ from human knees in terms of physiologic joint pressures and kinematics. Optimal implant position was simulated by physiological joint pressure during the testing, however the perfect implant position (flat or recessed to the surrounded cartilage) itself was not investigated.

## Conclusion

In this biomechanical set-up, an optimally positioned FMI (physiological contact pressure) lead to defects on the opposing cartilage after just six hours of cyclic loading. In the present set-up hyaluronic acid did not provide a beneficial effect, in fact, samples tested in hyaluronic acid experienced more profound and deeper injury. For clinical practice, a thorough analysis of pre-existing defects on the cartilage as well as meniscus pathologies that will oppose a FMI is recommended when a FMI is being considered. Pre-existing tibial cartilage defects and previous meniscectomy might be considered contraindication for implantation of a FMI. Nevertheless, for middle aged patients with focal cartilage defects, FMI might be a suitable treatment option.

## Data Availability

The datasets used and/or analysed during the current study are available from the corresponding author on reasonable request.

## Abbreviations

FMI: Focal metallic implant
MMA: Methyl methacrylate
OATS: Osteochondral autograft transfer
OA: Osteoarthritis
PRO: Patient reported outcome
TKA: Total knee arthroplasty

## References

[1] Allaire R, Muriuki M, Gilbertson L, Harner CD (2008). Biomechanical consequences of a tear of the posterior root of the medial meniscus. Similar to total meniscectomy. J Bone Joint Surg Am.

[2] Arno S, Bell CP, Uquillas C, Borukhov I, Walker PS (2015). Tibiofemoral contact mechanics following a horizontal cleavage lesion in the posterior horn of the medial meniscus. J Orthop Res.

[3] Årøen A, Løken S, Heir S, Alvik E, Ekeland A, Granlund OG (2004). Articular cartilage lesions in 993 consecutive knee arthroscopies. Am J Sports Med.

[4] Becher C, Huber R, Thermann H, Ezechieli L, Ostermeier S, Wellmann M (2011). Effects of a surface matching articular resurfacing device on tibiofemoral contact pressure: results from continuous dynamic flexion–extension cycles. Arch Orthop Trauma Surg.

[5] Becher C, Huber R, Thermann H, Paessler HH, Skrbensky G (2008). Effects of a contoured articular prosthetic device on tibiofemoral peak contact pressure: a biomechanical study. Knee Surg Sports Traumatol Arthrosc.

[6] Becher C, Huber R, Thermann H, Tibesku CO, von Skrbensky G (2009). Tibiofemoral contact mechanics with a femoral resurfacing prosthesis and a non-functional meniscus. Clin Biomech.

[7] Becher C, Kalbe C, Thermann H, Paessler HH, Laprell H, Kaiser T (2011). Minimum 5-year results of focal articular prosthetic resurfacing for the treatment of full-thickness articular cartilage defects in the knee. Arch Orthop Trauma Surg.

[8] Biswal S, Hastie T, Andriacchi TP, Bergman GA, Dillingham MF, Lang P (2002). Risk factors for progressive cartilage loss in the knee: a longitudinal magnetic resonance imaging study in forty-three patients. Arthritis Rheum.

[9] Bollars P, Bosquet M, Vandekerckhove B, Hardeman F, Bellemans J (2012). Prosthetic inlay resurfacing for the treatment of focal, full thickness cartilage defects of the femoral condyle: a bridge between biologics and conventional arthroplasty. Knee Surg Sports Traumatol Arthrosc.

[10] Christoforakis J, Pradhan R, Sanchez-Ballester J, Hunt N, Strachan RK (2005). Is there an association between articular cartilage changes and degenerative meniscus tears?. Arthroscopy.

[11] Curl WW, Krome J, Gordon ES, Rushing J, Smith BP, Poehling GG (1997). Cartilage injuries: a review of 31,516 knee arthroscopies. Arthroscopy.

[12] Custers R, Dhert W, Saris D, Verbout A, van Rijen M, Mastbergen S (2010). Cartilage degeneration in the goat knee caused by treating localized cartilage defects with metal implants. Osteoarthr Cartil.

[13] Custers R, Saris D, Dhert W, Verbout A, Van Rijen M, Mastbergen S (2009). Articular cartilage degeneration following the treatment of focal cartilage defects with ceramic metal implants and compared with microfracture. J Bone Joint Surg.

[14] Custers RJ, Creemers LB, van Rijen MH, Verbout AJ, Saris DB, Dhert WJ (2009). Cartilage damage caused by metal implants applied for the treatment of established localized cartilage defects in a rabbit model. J Orthop Res.

[15] Custers RJH, Dhert WJA, van Rijen MHP, Verbout AJ, Creemers LB, Saris DBF (2007). Articular damage caused by metal plugs in a rabbit model for treatment of localized cartilage defects. Osteoarthr Cartil.

[16] Davies-Tuck M, Wluka A, Wang Y, Teichtahl A, Jones G, Ding C (2008). The natural history of cartilage defects in people with knee osteoarthritis. Osteoarthr Cartil.

[17] Démarteau O, Pillet L, Inaebnit A, Borens O, Quinn TM (2006). Biomechanical characterization and in vitro mechanical injury of elderly human femoral head cartilage: comparison to adult bovine humeral head cartilage. Osteoarthr Cartil.

[18] Ethgen O, Bruyere O, Richy F, Dardennes C, Reginster J-Y (2004). Health-related quality of life in total hip and total knee arthroplasty. J Bone Joint Surg.

[19] Everhart JS, Abouljoud MM, Kirven JC, Flanigan DC (2019). Full-thickness cartilage defects are important independent predictive factors for progression to Total knee Arthroplasty in older adults with minimal to moderate osteoarthritis: data from the osteoarthritis initiative. J Bone Joint Surg Am.

[20] Fischer AH, Jacobson KA, Rose J, Zeller R. "Hematoxylin and eosin staining of tissue and cell sections." CSH Protoc. 2008. p. pdb.prot4986. 10.1101/pdb.prot4986 21356829

[21] Hangody L, Fules P (2003). Autologous osteochondral mosaicplasty for the treatment of full-thickness defects of weight-bearing joints: ten years of experimental and clinical experience. J Bone Joint Surg Am.

[22] Heck DA, Robinson RL, Partridge CM, Lubitz RM, Freund DA (1998). Patient outcomes after knee replacement. Clin Orthop Relat Res.

[23] Hjelle K, Solheim E, Strand T, Muri R, Brittberg M (2002). Articular cartilage defects in 1,000 knee arthroscopies. Arthroscopy.

[24] Kirker-Head CA, Van Sickle DC, Ek SW, McCool JC (2006). Safety of, and biological and functional response to, a novel metallic implant for the management of focal full-thickness cartilage defects: preliminary assessment in an animal model out to 1 year. J Orthop Res.

[25] Koh JL, Yi SJ, Ren Y, Zimmerman TA, Zhang L-Q (2016). Tibiofemoral contact mechanics with horizontal cleavage tear and resection of the medial meniscus in the human knee. JBJS.

[26] Kreuz PC, Erggelet C, Steinwachs MR, Krause SJ, Lahm A, Niemeyer P (2006). Is microfracture of chondral defects in the knee associated with different results in patients aged 40 years or younger?. Arthroscopy.

[27] Marcacci M, Kon E, Zaffagnini S, Iacono F, Neri MP, Vascellari A (2005). Multiple osteochondral arthroscopic grafting (mosaicplasty) for cartilage defects of the knee: prospective study results at 2-year follow-up. Arthroscopy.

[28] Martinez-Carranza N, Berg HE, Hultenby K, Nurmi-Sandh H, Ryd L, Lagerstedt AS (2013). Focal knee resurfacing and effects of surgical precision on opposing cartilage. A pilot study on 12 sheep. Osteoarthr Cartil.

[29] Martinez-Carranza N, Ryd L, Hultenby K, Hedlund H, Nurmi-Sandh H, Lagerstedt AS, et al. Treatment of full thickness focal cartilage lesions with a metallic resurfacing implant in a sheep animal model, 1 year evaluation. Osteoarthritis Cartilage. 2015. 10.1016/j.joca.2015.09.009. 10.1016/j.joca.2015.09.009 26403063

[30] McAdams TR, Mithoefer K, Scopp JM, Mandelbaum BR (2010). Articular cartilage injury in athletes. Cartilage.

[31] Meachim G (1972). Light microscopy of Indian ink preparations of fibrillated cartilage. Ann Rheum Dis.

[32] Meehan JP, Danielsen B, Kim SH, Jamali AA, White RH (2014). Younger age is associated with a higher risk of early periprosthetic joint infection and aseptic mechanical failure after total knee arthroplasty. J Bone Joint Surg Am.

[33] Milz S, Putz R (1994). Quantitative morphology of the subchondral plate of the tibial plateau. J Anat.

[34] Moore AC, Burris DL (2015). Tribological and material properties for cartilage of and throughout the bovine stifle: support for the altered joint kinematics hypothesis of osteoarthritis. Osteoarthr Cartil.

[35] Patel AM, Spector M (1997). Tribological evaluation of oxidized zirconium using an articular cartilage counterface: a novel material for potential use in hemiarthroplasty. Biomaterials.

[36] Shelbourne KD, Jari S, Gray T (2003). Outcome of untreated traumatic articular cartilage defects of the knee: a natural history study. J Bone Joint Surg Am.

[37] Spahn G, Wittig R (2003). Spannungs-und Bruchverhalten des gesunden Gelenkknorpels unter axialer Belastung. Eine biomechanische Untersuchung Zentralblatt für Chirurgie.

[38] Steadman JR, Briggs KK, Rodrigo JJ, Kocher MS, Gill TJ, Rodkey WG (2003). Outcomes of microfracture for traumatic chondral defects of the knee: average 11-year follow-up. Arthroscopy.

[39] Steadman JR, Rodkey WG, Singleton SB, Briggs KK (1997). Microfracture technique forfull-thickness chondral defects: technique and clinical results. Oper Tech Orthop.

[40] Venjakob AJ, Fohr P, Henke F, Tischer T, Sandmann GH, Blanke F, et al. Influence of sutures on cartilage integrity: do meniscus sutures harm cartilage? An experimental animal study. Arthroscopy. 2019. 10.1016/j.arthro.2018.11.040. 10.1016/j.arthro.2018.11.040 30745024

[41] Widuchowski W, Widuchowski J, Koczy B, Szyluk K (2009). Untreated asymptomatic deep cartilage lesions associated with anterior cruciate ligament injury: results at 10- and 15-year follow-up. Am J Sports Med.

